# Chemically Synthesized *Alcaligenes* Lipid A as an Adjuvant to Augment Immune Responses to *Haemophilus Influenzae* Type B Conjugate Vaccine

**DOI:** 10.3389/fphar.2021.763657

**Published:** 2021-10-22

**Authors:** Zilai Liu, Koji Hosomi, Atsushi Shimoyama, Ken Yoshii, Xiao Sun, Huangwenxian Lan, Yunru Wang, Haruki Yamaura, Davie Kenneth, Azusa Saika, Takahiro Nagatake, Hiroshi Kiyono, Koichi Fukase, Jun Kunisawa

**Affiliations:** ^1^ Laboratory of Vaccine Materials, Center for Vaccine and Adjuvant Research, and Laboratory of Gut Environmental System, National Institutes of Biomedical Innovation, Health, and Nutrition (NIBIOHN), Ibaraki, Japan; ^2^ Graduate School of Pharmaceutical Sciences, Osaka University, Suita, Japan; ^3^ Graduate School of Science, Osaka University, Toyonaka, Japan; ^4^ Graduate School of Medicine, Osaka University, Suita, Japan; ^5^ International Research and Development Center for Mucosal Vaccines, The Institute of Medical Science, The University of Tokyo, Tokyo, Japan; ^6^ Division of Gastroenterology, Department of Medicine, University of California San Diego (UCSD), San Diego, CA, United States; ^7^ Chiba University (CU)-UCSD Center for Mucosal Immunology, Allergy and Vaccines (cMAV), UCSD, San Diego, CA, United States; ^8^ Future Medicine Education and Research Organization, Chiba University, Chiba, Japan; ^9^ Division of Mucosal Immunology, IMSUT Distinguished Professor Unit, The Institute of Medical Science, The University of Tokyo, Tokyo, Japan; ^10^ Graduate School of Medicine, Kobe University, Kobe, Japan; ^11^ Research Organization for Nano and Life Innovation, Waseda University, Tokyo, Japan; ^12^ Graduate School of Dentistry, Osaka University, Suita, Japan

**Keywords:** alcaligenes, lipid A, adjuvant, TI antigen, haemophilus influenzae type B

## Abstract

We previously identified *Alcaligenes* spp. as a commensal bacterium that resides in lymphoid tissues, including Peyer’s patches. We found that *Alcaligenes*-derived lipopolysaccharide acted as a weak agonist of Toll-like receptor four due to the unique structure of lipid A, which lies in the core of lipopolysaccharide. This feature allowed the use of chemically synthesized *Alcaligenes* lipid A as a safe synthetic vaccine adjuvant that induces Th17 polarization to enhance systemic IgG and respiratory IgA responses to T-cell–dependent antigens (e.g., ovalbumin and pneumococcal surface protein A) without excessive inflammation. Here, we examined the adjuvant activity of *Alcaligenes* lipid A on a *Haemophilus* influenzae B conjugate vaccine that contains capsular polysaccharide polyribosyl ribitol phosphate (PRP), a T-cell–independent antigen, conjugated with the T-cell–dependent tetanus toxoid (TT) antigen (i.e., PRP-TT). When mice were subcutaneously immunized with PRP alone or mixed with TT, *Alcaligenes* lipid A did not affect PRP-specific IgG production. In contrast, PRP-specific serum IgG responses were enhanced when mice were immunized with PRP-TT, but these responses were impaired in similarly immunized T-cell—deficient nude mice. Furthermore, TT-specific—but not PRP-specific—T-cell activation occurred in mice immunized with PRP-TT together with *Alcaligenes* lipid A. In addition, coculture with *Alcaligenes* lipid A promoted significant proliferation of and enhanced antibody production by B cells. Together, these findings suggest that *Alcaligenes* lipid A exerts an adjuvant activity on thymus-independent Hib polysaccharide antigen in the presence of a T-cell–dependent conjugate carrier antigen.

## Introduction

Host immunity includes both innate and adaptive phases for the induction of antigen-specific immune responses. In general, the innate phase, a beginning of immune response reacts foreign antigen or pathogen in prompt manner using the pattern-recognition system (e.g., toll-like receptors [TLRs]), which leads to the activation of the adaptive immune response recognizes and eliminates pathogens specifically (Netea et al., 2019). Vaccines must use the host immune sequence of innate and adaptive phases for effectively promote the induction of an antigen-specific defense especially during the adaptive immune response (Messina et al., 2019). The activation of adaptive immunity involves antigen-presenting cells (APCs), such as dendritic cells (DCs), a key immune cell bridging the innate and adaptive phases of host immunity. For example, DCs can recognize microbial components (e.g., lipopolysaccharide [LPS]) through pattern-recognition receptors such as Toll-like receptors (Lipscomb and Masten, 2002), which induce the secretion of immune enhancing cytokines and promote antigen processing and presentation for the initiation and enhancement of antigen-specific immune responses (Lee and Iwasaki, 2007).

Stimulation by commensal bacteria is required for the development and maturation of host immunity (Zheng et al., 2020). We previously showed that the commensal bacterium *Alcaligenes* specifically resides within Peyer’s patches, a well characterized mucosa-associated lymphoid tissue for the initiation of antigen-specific immune responses in the intestine (Obata et al., 2010; Kunisawa and Kiyono, 2012). *Alcaligenes* organisms are taken up by DCs and promote the production of antibody-enhancing cytokines, including interleukin 6 (IL-6), thus leading to an elevated IgA antibody response in the intestine (Obata et al., 2010; Sato et al., 2013). In addition, compared with non-symbiotic *Escherichia coli*, symbiotic *Alcaligenes* have low inflammatory activity, which is explained at least partly by the unique features of its LPS (Fung et al., 2016; Shibata et al., 2018; Hosomi et al., 2020).

Several lines of evidence suggest that the structure of lipid A, which lies within the core of the LPS molecule, is related to its activity as a TLR4 ligand (Chandler and Ernst, 2017; Shimoyama et al., 2021). Compared with *E. coli*-derived lipid A, *Alcaligenes*-derived lipid A has shorter acyl chains that are modified with several functional groups, leading to appropriate activation of host immunity without excessive inflammation (Shimoyama et al., 2021). These characteristics prompted us to evaluate *Alcaligenes*-derived LPS and lipid A as a new and safe adjuvant candidate. Indeed, we found that both purified *Alcaligenes* LPS and chemically synthesized lipid A enhanced antibody production and Th17 responses to systemically or nasally immunized antigens (i.e., ovalbumin and pneumococcal surface protein A [PspA], a surface virulence factor of *Streptococcus pneumoniae*) (Wang et al., 2020; Yoshii et al., 2020; Wang et al., 2021).

B-cell responses are divided into two types, which differ regarding their need for T-cell involvement. T-cell–independent (TI) antigens induce rapid but short-lived production of IgM (Nutt et al., 2015). Because TI antigens, which include most polysaccharides, cannot be presented to T cells through major histocompatibility complex (MHC) class II (Mond et al., 1995), B-cell development and IgG class switching cannot be induced without input from T cells. In contrast, during T-cell–dependent (TD) B-cell responses, T cells are activated via their interaction with APCs through receptor pairing to MHC molecules and various costimulatory molecules (Mond et al., 1995).

*Haemophilus influenzae* type B (Hib), a Gram-negative pathogenic bacterium, is a frequent cause of bacterial meningitis among children (Anderson et al., 1977). The polyribosyl ribitol phosphate (PRP) of Hib has been used as the antigen for vaccines against Hib. Because PRP is a TI antigen, commercially available vaccines (e.g., ActHib) include a modified PRP to which a TD carrier protein antigen (e.g., tetanus toxoid) has been conjugated, to enhance the immunogenicity of PRP (Guttormsen et al., 1999; Kelly et al., 2004).

Although our previous studies (Wang et al., 2020; Yoshii et al., 2020; Wang et al., 2021) demonstrated that *Alcaligenes* lipid A is an effective adjuvant for TD antigens such as ovalbumin and PspA, whether it also efficiently boosts the antigenicity of TI antigens remained unclear. Here we aimed to extend the application of *Alcaligenes* lipid A by determining its adjuvanticity on a *Haemophilus* B conjugate vaccine as an example TI antigen–based conjugate vaccine.

## Materials and Methods

### Mice

Because Hib vaccines are mainly used in infants, female BALB/c and nu/nu BALB/c mice were obtained after finishing lactation (age, 4 weeks, CLEA Japan, Tokyo, Japan) and kept for 1 week before experiments were initiated. All animal experiments were conducted in accordance with the Animal Care and Use Committee guidelines of the National Institutes of Biomedical Innovation, Health, and Nutrition (NIBIOHN) and the Committee on the Ethics of Animal Experiments of NIBIOHN (approval nos. DS25-2 and DS25-3).

### Preparation of *Alcaligenes* Lipid A and PRP-Tyramine

*Alcaligenes* lipid A was chemically synthesized as previously described (Shimoyama et al., 2021), dissolved in dimethyl sulfoxide (Nacalai Tesque, Tokyo, Japan), and stored at −30°C.

For use as the coating antigen in enzyme-linked immunosorbent assays (ELISAs), PRP (National Institute for Biological Standards and Control, United Kingdom) was coupled to tyramine as follows. Briefly, 5 mg of PRP was dissolved in 10 ml of 0.01 N NaOH (Nacalai Tesque); we then added 65 μl of acetonitrile (FUJIFILM) containing 65 mg of cyanogen bromide (FUJIFILM) to the NaOH solution. The pH of the solution was maintained at 10.8 with 0.1 N NaOH and incubated at room temperature for 10 min. After PRP was activated, 1 ml of 0.5 M NaHCO_3_ (Nacalai Tesque) containing 50 mg of tyramine hydrochloride (FUJIFILM) was added to the solution, and the pH was adjusted to 8.5 with 0.1 N HCl (FUJIFILM). The solution was transferred into dialysis bags (Sigma-Aldrich, St. Louis, MO, United States) and dialyzed against distilled water at 4°C for 24 h followed by phosphate-buffered saline (PBS) at 4°C for 24 h (Kaplan et al., 1983; Barra et al., 1988). The coupled PRP-tyramine was stored at −80°C until use.

### Immunization

Mice were anesthetized with isoflurane (FUJIFILM Wako Pure Chemical, Osaka, Japan) and then subcutaneously immunized with a total volume of 200 μl PBS containing either 0.01 μg of the Hib capsular polysaccharide PRP, 0.01 μg of PRP plus 0.024 μg of tetanus toxoid (TT) (EMD Millipore, Burlington, MA, United States), *Haemophilus* B PRP–TT conjugate vaccine (ActHIB; Sanofi, Tokyo, Japan) equivalent to 0.01 μg of PRP or 1 μg of PRP with or without 1 μg of *Alcaligenes* lipid A (Wang et al., 2020), or PBS only. Mice received three immunizations at 1-week intervals. One week after the final immunization, blood was harvested from the mice and kept on ice until centrifuged at 4°C, 3,000 × *g* for 10 min. The serum was transferred into a fresh tube and stored at −80°C.

### Detection of PRP and TT-specific Antibodies by ELISA

The production of PRP-specific and TT-specific antibodies was detected by ELISA. Briefly, 96-well immunoplates (Thermo Fisher Scientific, Waltham, MA, United States) were coated with 5 μg/ml PRP-tyramine or 0.1 μg/ml TT in PBS at 4°C overnight. After the coating solution was removed, the plates were saturated with 1% bovine serum albumin (Nacalai Tesque) dissolved in PBS for 2 h at room temperature. Plates were then rinsed 3 times with wash buffer (PBS containing 0.05% Tween 20 [Nacalai Tesque]). Each well then received mouse serum (2-fold serially diluted in PBS containing 0.05% Tween 20 and 1% bovine serum albumin), and the plates were incubated at room temperature for 2 h. The plates were then again washed 3 times with wash buffer; goat anti-mouse IgG, IgG1, IgG2a, IgG3 antibody conjugated with horseradish peroxidase (SouthernBiotech; diluted 1:4,000 in PBS containing 1% bovine serum albumin and 0.05% Tween 20) was added to each well; and the plates were incubated at room temperature for 1 h. The plates again were washed 3 times with wash buffer; tetramethylbenzidine peroxidase substrate (SeraCare Life Sciences, Milford, MA, United States) was added to the plates; and the plates were incubated at room temperature for 2 min, after which 0.5 N HCl (Nacalai Tesque) was added to each well. The absorbance of samples at 450 nm (OD_450_) was measured by using an iMarkTM Microplate Absorbance Reader (Bio-Rad Laboratories, Hercules, CA, United States).

### T-Cell Assay

At 1 week after the final immunization, the spleens from immunized mice were harvested, homogenized, and then filtered through 100-μm cell strainers (Corning, New York, NY, United States) separately. These single-cell suspensions were treated with 1 ml of red blood cell lysis buffer [10 mM NaHCO_3_, 1 mM EDTA-2Na·2H_2_O (Dojindo Molecular Technologies, Kumamoto, Japan), 0.15 M NH_4_Cl (Nacalai Tesque)] for 1 min at room temperature. Splenic CD4^+^ T cells were purified by using a magnetic cell separation system and anti-mouse CD4 (L3T4) magnetic beads (Miltenyi Biotec, Bergisch Gladbach, Germany) and MS columns (Miltenyi Biotec). Purified CD4^+^ T cells were resuspended in RPMI medium (Sigma-Aldrich, St. Louis, MO, United States) containing 10% fetal bovine serum (Gibco, Thermo Fisher Scientific), 1 mM sodium pyruvate solution (Nacalai Tesque), 1% penicillin–streptomycin mixed solution (Nacalai Tesque), and 0.1% 2-mercaptoethanol (Gibco, Thermo Fisher Scientific) and were seeded at a concentration of 2 × 10^5^ cells/well into 96-well plates (Nunc 96-Well, Nunclon Delta-Treated, U-Shaped-Bottom Microplates, Thermo Fisher Scientific). Each well also received splenic APCs (2 × 10^4^ cells/well) from unimmunized mice that had been treated with 30 Gy of ionizing radiation (MBR-1520R-4, Hitachi, Tokyo, Japan). The purified CD4^+^ T cells mixed with APCs were incubated in the presence or absence of 5 μg/ml of TT or 2.08 μg/ml of PRP at 37°C in 5% CO_2_. After 4 days of incubation, live T cells were counted by using CyQUANTTM Direct Cell Proliferation Assay Kits (Invitrogen, Thermo Fisher Scientific). Cytokines in the supernatant was measured by the BD^™^ Cytometric Bead Array (CBA) Mouse Th1/Th2/Th17 Cytokine Kit (BD Biosciences, San Jose, CA, United States) and analyzed with a MACSQuant® Analyzer (Miltenyi Biotec).

### Coculture of B Cells With *Alcaligenes* Lipid A and Measurement of IgG Production

Spleens from naïve mice were homogenized and filtered through 100-μm cell strainers. The suspensions were treated with 1 ml of red blood cell lysis buffer for 1 min at room temperature. Splenic B220^+^cells were purified by using a magnetic cell separation system with anti-mouse CD45R (B220) magnetic beads (Miltenyi Biotec) and LS Columns (Miltenyi Biotec). The B cells were then seeded (10^5^ cells/well) into 96-well plates without or with 100 ng/ml of *Alcaligenes* lipid A and incubated at 37°C in 5% CO_2_. After the 5-days incubation, live B cells were counted by using CyQUANTTM Direct Cell Proliferation Assay Kits.

The total IgG contents in the B-cell culture supernatant were measured by using antigen-specific ELISAs. Briefly, 96-well immunoplates were coated with 2 μg/ml goat anti-mouse Ig (SouthernBiotech). After the plates were washed, dilutions of culture supernatant and standard antibody (unconjugated mouse IgG, SouthernBiotech) were added to wells, and plates were incubated at room temperature for 2 h. The plates were washed again; wells were treated with goat anti-mouse IgG antibodies conjugated with horseradish peroxidase and tetramethylbenzidine peroxidase substrate; and absorbance at 450 nm was determined.

### Statistical Analysis

Data are presented as mean ±1 SD. Statistical analyses were performed by using Student’s *t*-test and one-way ANOVA with Tukey’s multiple comparison test (PRISM 8.4.3, GraphPad Software, San Diego, CA, United States).

## Results

### *Alcaligenes* Lipid A Enhances Both PRP- and TT-specific IgG Responses After Immunization With *Haemophilus* B Conjugate Vaccine

**FIGURE 1:**
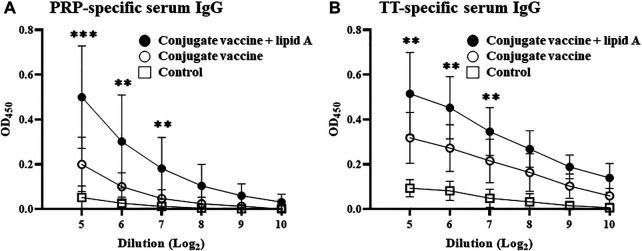
*Alcaligenes* lipid A enhanced antigen-specific IgG production in *Haemophilus* B conjugate vaccination. Mice were immunized subcutaneously with PBS (control group) or Haemophilus B conjugate vaccine containing 0.01 μg of PRP with or without 1 μg of *Alcaligenes* lipid A. Serum was collected 1 week after the final immunization, and the levels of **(A)** PRP-specific IgG and **(B)** TT-specific IgG were measured by ELISA (*n* = 11/group). The results shown are presented as mean ±1 SD. Data are representative of two independent experiments, and statistical significance was evaluated by using one-way ANOVA (**, *p* < 0.01; ***, *p* < 0.001; the asterisks represent the significant difference between two experimental groups).

When PRP is conjugated to a protein, such as TT, the complex acts as a TD antigen and thus induces PRP-specific IgG production (Guttormsen et al., 1999; Kelly et al., 2004). We therefore first examined whether *Alcaligenes* lipid A enhances the immune response against the conjugated PRP of the *Haemophilus* B conjugate vaccine. Consistent with a previous study (Schneerson et al., 1980), PRP-specific IgG production was induced in mice immunized with the *Haemophilus* B conjugate vaccine compared with PBS (as a control). Specifically, mice immunized with *Haemophilus* B conjugate vaccine in the presence of *Alcaligenes* lipid A had higher levels of PRP-specific serum IgG than did mice immunized with *Haemophilus* B conjugate vaccine alone at different antigen doses of 0.01 and 1 μg of PRP (Figures 1A; Supplementary Figure S1). Among IgG subtypes, higher levels of IgG3 were detected in groups immunized with *Alcaligenes* lipid A (Supplementary Figure S2C). In addition, mice immunized with *Haemophilus* B conjugate vaccine plus *Alcaligenes* lipid A had higher levels of TT-specific serum IgG (Figure 1B). These results show that *Alcaligenes* lipid A can enhance the production of IgG against PRP, a TI antigen, when PRP is conjugated to TT.

### Conjugation of PRP to the TT Carrier Protein is Necessary for *Alcaligenes* Lipid A–Mediated Enhancement of a PRP-Specific IgG Response

**FIGURE 2:**
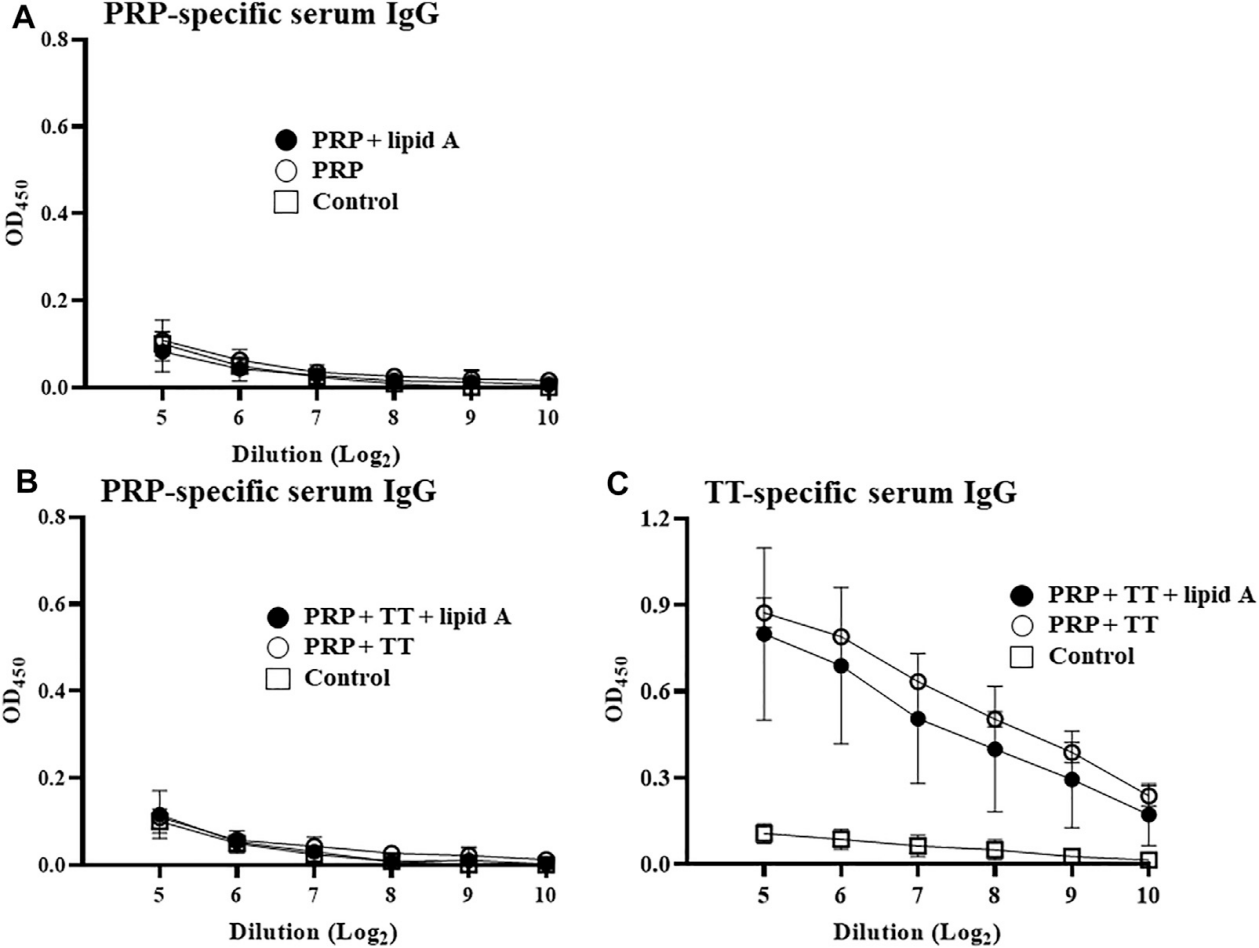
Conjugation of PRP to TT carrier protein is essential for enhancement of PRP-specific IgG production by *Alcaligenes* lipid A. **(A)** Mice were immunized subcutaneously with PBS (control group) or 0.01 μg of unconjugated PRP with or without 1 μg of *Alcaligenes* lipid A. **(B)** Mice were immunized subcutaneously with PBS (control group) or 0.01 μg of unconjugated PRP plus 0.024 μg of TT and with or without 1 μg of *Alcaligenes* lipid A. Serum was collected 1 week after the final immunization, and the level of PRP-specific IgG was measured by ELISA (experimental group, *n* = 5; control group, *n* = 4). **(C)** Mice were immunized subcutaneously with PBS (control group) or 0.01 μg of unconjugated PRP plus 0.024 μg of TT and with or without 1 μg of Alcaligenes lipid A. Serum was collected 1 week after the final immunization, and the level of TT-specific IgG was measured by ELISA (n=5/group). Data are representative of two independent experiments and are presented as mean ±1 SD.

To verify the importance of the conjugation of carbohydrate antigen PRP to protein career TT for the adjuvant activity of *Alcaligenes* lipid A in the enhanced PRP-specific IgG production, we next immunized mice with either PRP only or mixed (no physical coupling) with TT in the presence or absence with *Alcaligenes* lipid A. Neither immunization with PRP alone nor with PRP plus lipid A induced an IgG response (Figure 2A). Furthermore, no PRP-specific IgG response was detected in mice immunized with both antigens (PRP mixed with TT) even with *Alcaligenes* lipid A (Figure 2B); meanwhile, TT-specific IgG response was not enhanced (Figure 2C). These findings indicate that *Alcaligenes* lipid A enhances IgG production against PRP only when PRP is conjugated with TT.

### T Cells are Required for the Induction of a PRP-Specific IgG Response

**FIGURE 3:**
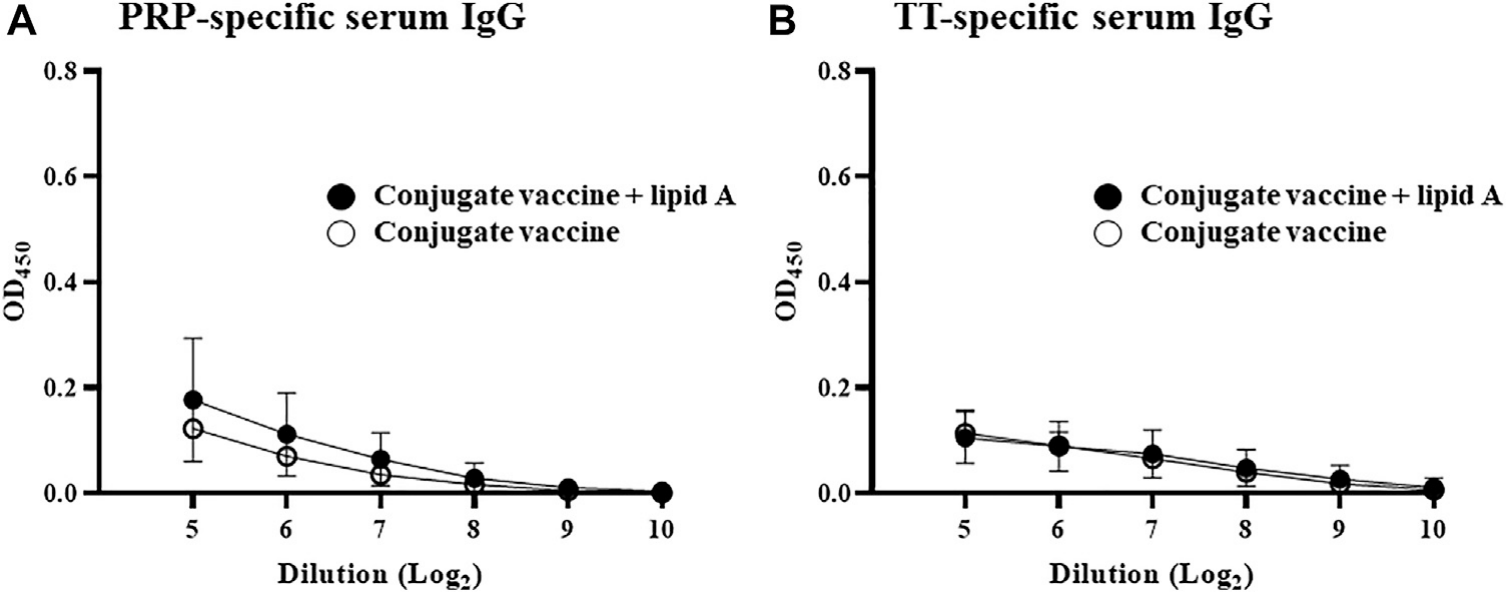
T cells are required for Alcaligenes lipid A–promoted PRP-specific IgG production. T cell–deficient nude mice were immunized subcutaneously with Haemophilus B conjugate vaccine with or without 1 μg of Alcaligenes lipid A. Serum was collected 1 week after the final immunization, and the levels of **(A)** PRP-specific IgG and **(B)** TT-specific IgG were measured by ELISA. Data are representative of two independent experiments and are presented as mean ± 1 SD (n = 4/group).

We found that conjugation of PRP with TT is necessary for the induction and augmentation of PRP-specific IgG response by *Alcaligenes* lipid A. These findings led us to examine the importance of T cells in *Alcaligenes* lipid A-mediated enhancement of antigen-specific IgG production. Immunization of nude mice, which have a deteriorated or absent thymus and thus lack T cells, with *Haemophilus* B conjugate vaccine with or without *Alcaligenes* lipid A induced scant PRP-specific IgG production (Figure 3A) and no TT-specific IgG response (Figure 3B). These results show that *Haemophilus* B conjugate vaccine-induced IgG responses to either PRP or TT require T cells and that *Alcaligenes* lipid A cannot augment these IgG responses in the absence of T cells.

### T Cells are Induced by *Haemophilus* B Conjugate Vaccine but are not Enhanced by *Alcaligenes* Lipid A

**FIGURE 4:**
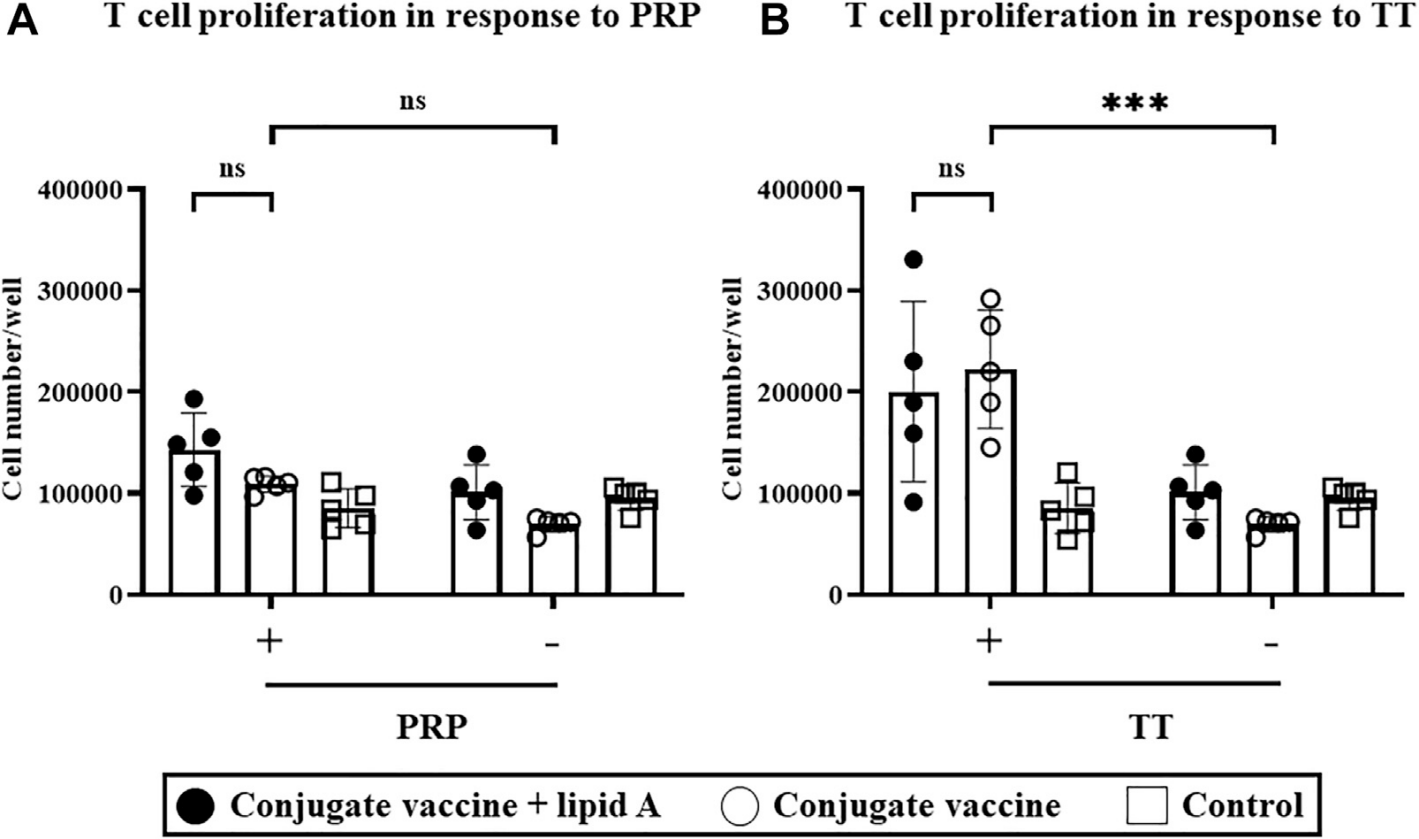
*Alcaligenes* lipid A has no effect on the TT-specific T-cell response. Mice were immunized subcutaneously with *Haemophilus* B conjugate vaccine with or without 1 μg of *Alcaligenes* lipid A; control mice were immunized with PBS. Splenic CD4^+^ cells were collected 1 week after the final immunization and stimulated with (+) or without (–) **(A)** 2.08 μg/ml PRP or **(B)** 5 μg/mL TT. After stimulation for 4 days, live CD4^+^ cells were counted. Data are representative of two independent experiments and are presented as mean ±1 SD. (*n* = 5/group), and statistical significance was evaluated by using one-way ANOVA (ns, not significant; ***, *p* < 0.001).

Since T cells are required for adjuvanticity of *Alcaligenes* lipid A to enhance antigen-specific antibody production in response to the *Haemophilus* B conjugate vaccine, we next investigated the effects of *Alcaligenes* lipid A on T cells. We isolated splenic CD4^+^ T cells from mice immunized with *Haemophilus* B conjugate vaccine with or without *Alcaligenes* lipid A and measured their ability to proliferate *ex vivo* upon stimulation with antigen (e.g., PRP and TT) in the presence of APCs. Stimulation with TT (Figure 4B)—but not PRP (Figure 4A)—increased T-cell counts in the immunized groups. However, including *Alcaligenes* lipid A at immunization did not further increase the number of T cells (Figure 4B). Cytokine analysis revealed that IL-17A was preferentially detected in the immunized groups (Supplementary Figure S3C). These results show that although the *Haemophilus* B conjugate vaccine induces a TT-specific T-cell response, concurrent immunization with *Alcaligenes* lipid A does not enhance it.

### *Alcaligenes* Lipid A Activates B Cells, Leading to Enhanced Cell Numbers and Antibody Production

**FIGURE 5:**
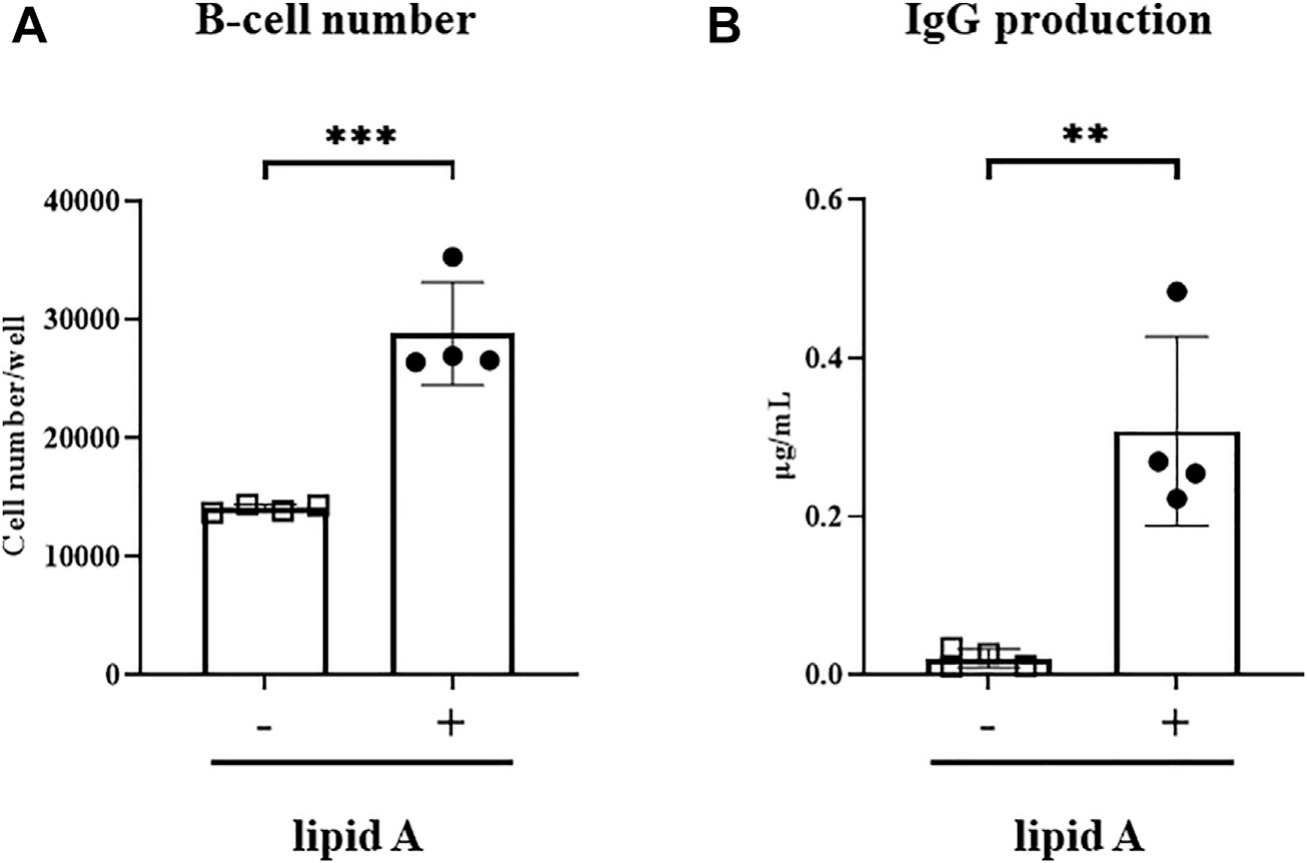
*Alcaligenes* lipid A directly activates B cells. Splenic B220^+^ cells were isolated from naive mice. After 4 days of culture with (+) or without (–) *Alcaligenes* lipid A, **(A)** live B cells were counted, and the **(B)** IgG content in the culture supernatant was measured. Data are representative of two independent experiments and are presented as mean ±1 SD, and statistical significance was evaluated by using Student’s *t*-test (*n* = 4/group; **, *p* < 0.01; ***, *p* < 0.001).

Given that *Alcaligenes* lipid A failed to enhance the T-cell response to the *Haemophilus* B conjugate vaccine, we wondered whether direct stimulation of B cells might lead to upregulation of IgG secretion. We therefore cocultured naïve splenic B220^+^ B cells with *Alcaligenes* lipid A. Treatment with *Alcaligenes* lipid A significantly increased the number of B cells (Figure 5A) and the amounts of IgG (Figure 5B) in culture supernatants. These results indicate that *Alcaligenes* lipid A directly promotes B-cell proliferation and antibody production.

## Discussion

We previously reported that *Alcaligenes* LPS acts as an TLR4 agonist, thereby enhancing antigen-specific immune responses without excessive inflammation and leading to the possibility of its use as a safe adjuvant (Shibata et al., 2018; Wang et al., 2021). LPS is mainly composed of lipid A, core oligosaccharide, and O antigen among which lipid A is considered to be the active site of LPS and major determinant of LPS activity. It is known that the structure of lipid A differs among bacteria, and we recently reported that *Alcaligenes* lipid A possesses hexa-acylated species that was composed of a bisphosphorylated glucosamine disaccharide backbone carrying 14:0 (3-OH) as primary and 12:0 (3-OH) and 10:0 as secondary fatty acids with distribution in a 3 + 3 symmetric fashion with respect to the disaccharide backbone, which were different from *E. coli* lipid A that has 4 + 2 asymmetry and is composed of 14:0 (3-OH) as primary and 14:0 and 12:0 as secondary fatty acids (Shimoyama et al., 2021) and could be used to enhance immune responses against T cell-dependent antigens (Wang et al., 2020; Yoshii et al., 2020). Current study extended our researches by demonstrating the efficacy of *Alcaligenes* lipid A as an adjuvant for a Hib vaccine that includes the TI antigen PRP. Specifically, *Alcaligenes* lipid A enhanced the PRP-specific IgG response when Hib PRP was conjugated to a TD antigen (i.e., TT) as a carrier protein. Together, our current findings indicate that *Alcaligenes* lipid A can enhance TI antigen–specific antibody production in the presence of TD antigen.

An unexpected finding in the current study was that *Alcaligenes* lipid A did not enhance the T cell-response to immunization (Figure 4), suggesting that the pathway through which *Alcaligenes* lipid A increases the IgG response differs from that through which it activates DCs and thus influences the T-cell response. One possible reason might be the way through which antigens are presented to T cells. For TD antigens, DCs act as the major APCs and present antigens to T cells for activation together with costimulatory molecules such as CD80 (Tai et al., 2018). Meanwhile, cytokines secreted by DCs also sense T cells, inducing different types of response (Terhune et al., 2013), which then activate B cells through cell-contact. In contrast, B cells recognize glycoconjugate antigens because of their carbohydrate portion and thus retrieve the entire antigen through B-cell receptors (Popi et al., 2016; Avci et al., 2019). After being processed, the peptide portion is presented to T cells via MHC II, thus activating T cells, which then secrete cytokines to activate B cells (Avci and Kasper, 2010; Avci et al., 2011). During the first type of response, DCs produce T-cell–activating cytokines, including IL-12 (which induces Th1 differentiation), IL-4 (Th2 differentiation), and IL-6, IL-23, and TGF-β (Th17 differentiation) (Kimura and Kishimoto, 2010; Terhune et al., 2013). In contrast, B cells, which might secrete only negligible amounts of other cytokines, produce considerable IL-6 (Chousterman and Swirski, 2015), suggesting that the T-cell response induced by B cells may differ from that induced by DCs. Hence, perhaps the way in which T cells are activated by APCs influences the subsequent T-cell response. Thus, *Alcaligenes* lipid A may preferentially activate B cells directly to yield adjuvanticity for the *Haemophilus* B conjugate vaccine.

TLR4 is expressed not only on APCs such as DCs (Vaure and Liu, 2014), which has been proved to be a target for *Alcaligenes* lipid A (Shibata et al., 2018) during responses induced by TD antigens such as PspA, but also on B cells (Vaure and Liu, 2014), thus suggesting at least two possible mechanisms through which *Alcaligenes* lipid A exerts its effects on antigen-specific IgG production. Regarding a first possibility of a direct effect of lipid A on B cells, coculture with *Alcaligenes* lipid A increased B-cell numbers and their ability to secrete IgG (Figure 5). These effects likely occurred through the TLR4 pathway. B-cell proliferation might involve the phosphatidylinositol 3-kinase signaling pathway (Venkataraman et al., 1999), which can be induced through TLR4 signaling (Dil and Marshall, 2009). In addition, due to upregulation of MyD88, B cells in germinal centers (GCs) show increased reactivity to TLR ligands, leading to enhanced proliferation and promotion of class-switching recombination; MyD88 facilitates B-cell differentiation into plasma cells (Rawlings et al., 2012). The second possible mechanism underlying the enhancement of IgG production in response to *Alcaligenes* lipid A is through effects on DC-mediated antigen-specific T-cell responses. Because PRP is a TI antigen (Guttormsen et al., 1999; Kelly et al., 2004), the induction of a PRP-specific IgG response required both T cells and conjugation of PRP with TT. Indeed, lacking the help from T cells, polysaccharide-activated B cells undergo apoptosis and thus fail to mount a PRP-specific IgG response (Cobb et al., 2004; Rappuoli, 2018; Rappuoli et al., 2019). The conjugation with TT may not only induce T cell-response but also affect the antigen uptake. It was reported that the TD portion of conjugate vaccine mediated their uptake by DCs, which will trigger the formation of GCs (Rappuoli, 2018). Meanwhile, our previous study demonstrated that *Alcaligenes* lipid A induced the formation of GCs (Yoshii et al., 2020) in which B cells activated through TLRs show higher viability (Rawlings et al., 2012). These findings collectively implicate a possibility that *Alcaligenes* lipid A may enhance the antibody production against conjugated vaccine directly through the contact to B cells and indirectly through the simultaneous induction of GCs. However, apart from the conjugation, the TT itself might also influence the response because, unexpectedly, when TT was co-administrated with PRP together with *Alcaligenes* lipid A, TT-specific response was not enhanced. The mechanism hasn’t been fully explained because the reaction of LPS-induced TT-specific response is not very representative (Mohammadi et al., 2014).

In general, vaccination with TI antigens induces IgM-mediated immunity only, and long-lasting IgG-mediated immunity has been difficult to achieve. Conjugation of TD carrier proteins to some TI antigens can induce class-switching (Avci et al., 2011). The resulting induction of IgG production has increased the efficacy of immune responses against various TI antigens to provide sufficient protection and even prevention in some cases (Cochi et al., 1985; Granoff et al., 1993). In the current study, the adjuvant activity of the *Alcaligenes* lipid A on the TI antigen in the *Haemophilus* B conjugate vaccine (i.e., PRP) presumably was mediated through the direct activation of B cells instead of via enhancement of T-cell responses. In addition to creating a conjugate vaccine that induces a sufficient T-cell response for the induction of class-switching to IgG, enhancing the proliferation of B cells and their IgG secretion will enhance immune responses to TI antigens. Thus, our current findings support the use of *Alcaligenes* lipid A as an adjuvant to augment and accelerate vaccine-induced immune responses.

In conclusion, *Alcaligenes* lipid A exerted adjuvant activity for a TI polysaccharide antigen only when it was conjugated to a TD carrier protein. The induction pathway for the TI antigen did not include enhancement of T-cell responses and thus differs from that of TD antigens (Wykes and Macpherson, 2000).

## Data Availability

The raw data supporting the conclusion of this article will be made available by the authors, without undue reservation.
