# Supplementary figures and images for: Chemically Synthesized Alcaligenes Lipid A as an Adjuvant to Augment Immune Responses to Haemophilus Influenzae Type B Conjugate Vaccine
Source: Front Pharmacol. 2021 Oct 22;12:763657. doi: 10.3389/fphar.2021.763657 (PMC8569242; doi:10.3389/fphar.2021.763657)

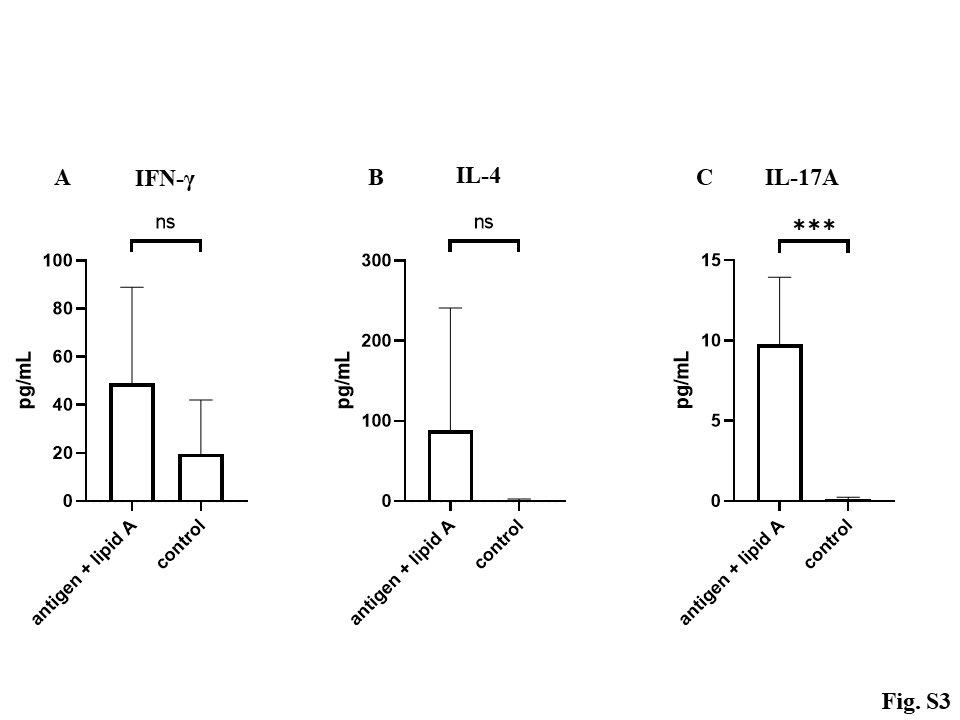

Supplement: Supplementary file 1 [file Image3.TIF]

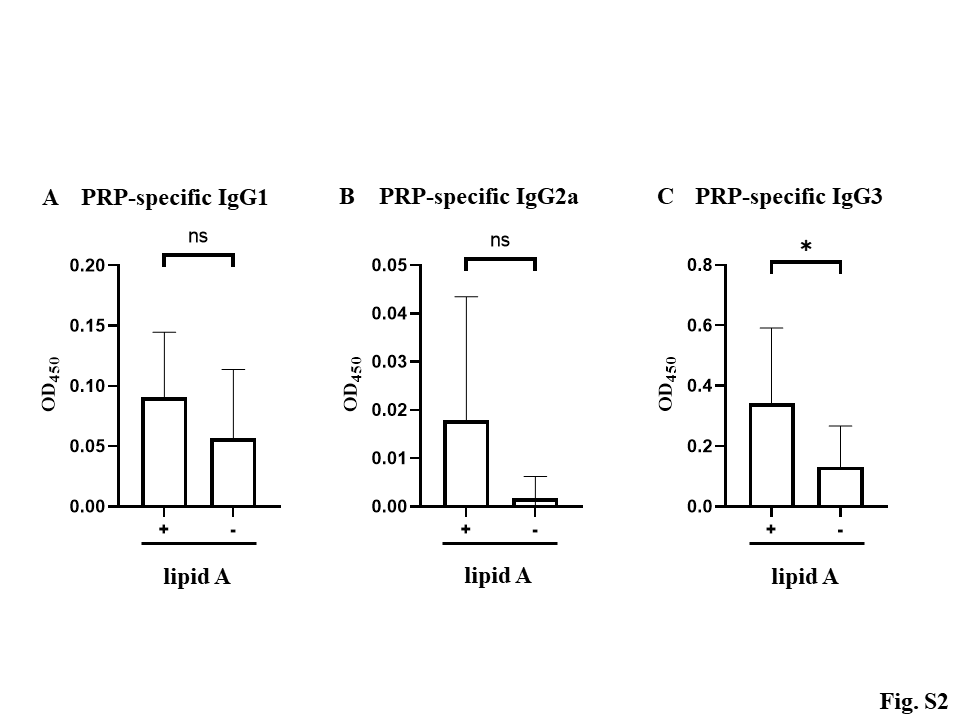

Supplement: Supplementary file 2 [file Image2.TIF]

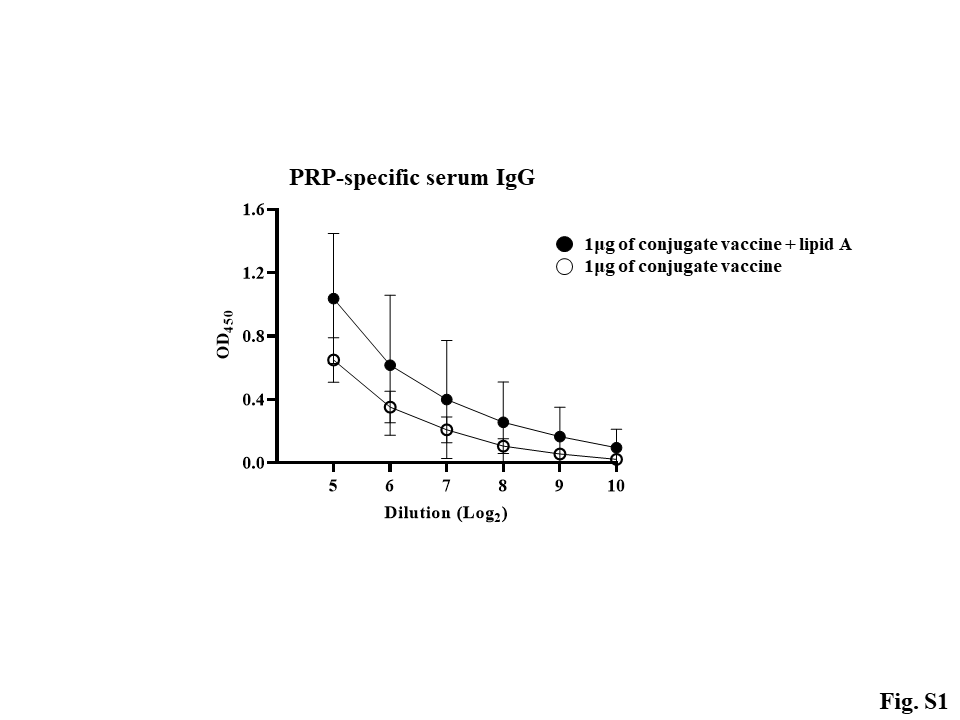

Supplement: Supplementary file 3 [file Image1.TIF]
